# *Salmonella* DIVA vaccine reduces disease, colonization and shedding due to virulent *S.* Typhimurium infection in swine

**DOI:** 10.1099/jmm.0.000482

**Published:** 2017-05-18

**Authors:** Bradley L Bearson, Shawn M. D Bearson, Brian W Brunelle, Darrell O Bayles, In Soo Lee, Jalusa D Kich

**Affiliations:** ^1^​ USDA/ARS/National Laboratory for Agriculture and the Environment, Ames, IA, 50011, USA; ^2^​ USDA/ARS/National Animal Disease Center, Ames, IA, 50010, USA; ^3^​ Hannam University, Daejeon, Republic of Korea; ^4^​ Embrapa Swine and Poultry, Concórdia, SC, Brazil

**Keywords:** *Salmonella*, DIVA, vaccine, swine, animal health, food safety

## Abstract

**Purpose:**

Non-host-adapted *Salmonella* serovars, including the common human food-borne pathogen *Salmonella enterica* serovar Typhimurium (*S.* Typhimurium), are opportunistic pathogens that can colonize food-producing animals without causing overt disease. Interventions against *Salmonella* are needed to enhance food safety, protect animal health and allow the differentiation of infected from vaccinated animals (DIVA).

**Methodology:**

An attenuated *S*. Typhimurium DIVA vaccine (BBS 866) was characterized for the protection of pigs following challenge with virulent *S.* Typhimurium. The porcine transcriptional response to BBS 866 vaccination was evaluated. RNA-Seq analysis was used to compare gene expression between BBS 866 and its parent; phenotypic assays were performed to confirm transcriptional differences observed between the strains.

**Results:**

Vaccination significantly reduced fever and interferon-gamma (IFNγ) levels in swine challenged with virulent *S*. Typhimurium compared to mock-vaccinated pigs. *Salmonella* faecal shedding and gastrointestinal tissue colonization were significantly lower in vaccinated swine. RNA-Seq analysis comparing BBS 866 to its parental *S.* Typhimurium strain demonstrated reduced expression of the genes involved in cellular invasion and bacterial motility; decreased invasion of porcine-derived IPEC-J2 cells and swimming motility for the vaccine strain was consistent with the RNA-Seq analysis. Numerous membrane proteins were differentially expressed, which was an anticipated gene expression pattern due to the targeted deletion of several regulatory genes in the vaccine strain. RNA-Seq analysis indicated that genes involved in the porcine immune and inflammatory response were differentially regulated at 2 days post-vaccination compared to pre-vaccination.

**Conclusion:**

Evaluation of the *S*. Typhimurium DIVA vaccine indicates that vaccination will provide both swine health and food safety benefits.

## Introduction

In many countries, *Salmonella* is endemic and often asymptomatic in swine production, thereby complicating the control of this opportunistic animal health and human food-borne pathogen in pigs [1]. The pervasive nature of *Salmonella* in swine production is highlighted by the frequent presence of the food-borne pathogen in breeding herds. For example, in the EU, the prevalence of *Salmonella* in breeding herds is up to 56 % [2]. A review of our sow-screening data from a small number of farms in the USA with high health status (including specific-pathogen-free farms) found that 95 % of sows had serum antibodies to *Salmonella* LPS and ~20 % were actively shedding the pathogen in their faeces prior to farrowing [3]. These data indicate that, during their lifetime, most sows are exposed to *Salmonella* and piglets from a subset of these sows will most likely be exposed to *Salmonella* within days of farrowing.

To protect swine against *Salmonella* colonization, on-farm interventions are needed. Developing *Salmonella* management strategies on farms is complicated by the dual goal of controlling disease in the animal while reducing subclinical *Salmonella* carriage and shedding for the protection of pork consumers. Production control strategies often involve a combinatorial approach that can include vaccination [2]. A recent review by Wales and Davies [4] highlights the current challenges for *Salmonella* vaccination in the swine industry. Two major hurdles for vaccines against *Salmonella* are avoiding interference with serological monitoring programs and demonstrating cross-protection against diverse *Salmonella* serovars with variable immunodominant antigens. We recently published a report detailing cross-protection against *Salmonella enterica* serovar Choleraesuis using our attenuated *S. enterica* serovar Typhimurium (*S*. Typhimurium) BBS 866 vaccine strain [5]. Furthermore, the rational design of the vaccine prevented interference with the established IDEXX Swine *Salmonella* Ab Test; thus, the vaccine strain can be used in swine without compromising the differentiation of infected from vaccinated animals (DIVA).

In the current study, our objectives were to compare the gene expression of the *S*. Typhimurium BBS 866 vaccine strain to the parental wild-type strain, evaluate vaccine protection against a virulent *S.* Typhimurium strain in swine, and transcriptionally profile the porcine response to the vaccine strain. Vaccination with *S*. Typhimurium BBS 866 reduced clinical symptoms (fever), IFNγ response, *Salmonella* faecal shedding and gastrointestinal tissue colonization in swine subsequently challenged with the wild-type *S*. Typhimurium strain. Gene expression comparisons identified 1579 genes differentially expressed between the *S*. Typhimurium parental and vaccine strains. Transcriptional profiling of the vaccinated swine identified differential expression of immune-related genes in response to the vaccine strain.

## Methods

### Bacterial strains

The attenuated *S.* Typhimurium DIVA vaccine strain BBS 866 (Δ*rybB* Δ*omrAB* Δ*micA* Δ*invR rfaH *::*neo*) was described in Bearson *et al*. [5]. Two wild-type *S.* Typhimurium strains (χ4232 and SB 377) were used during this study. *S.* Typhimurium strain χ4232 is the virulent parent of strain BBS 866. Strain SB 377 is a nalidixic acid-resistant derivative of virulent *S.* Typhimurium UK1 that was previously inoculated intranasally into a pig and re-isolated from the ileocecal lymph node 7 days after challenge.

### Swine trial

Twenty crossbred, conventionally raised, mixed-gender piglets from four *Salmonella*-faecal-negative sows were weaned at 12 days of age and shipped to the National Animal Disease Center, Ames, IA, USA. Siblings from each litter were divided and raised in two isolation rooms (*n*=10 pigs per room). Piglets were tested to ensure that they were faecal-negative for *Salmonella* spp. twice over a 2 week period using bacteriological culture with selective enrichment [6]. At 4 weeks of age, piglets received an intranasal inoculation of 1 ml PBS (mock-vaccinated) or PBS containing 1.0×10^10^ c.f.u. BBS 866. At 6 weeks of age, piglets received a booster of either PBS or PBS containing 4.0×10^9^ c.f.u. of BBS 866. At 9 weeks of age, all pigs were intranasally challenged with 1 ml PBS containing 2×10^8^ c.f.u. virulent SB 377 and monitored for clinical disease parameters and *Salmonella* faecal shedding over a 7 day period (see below). At day 7 following challenge, all pigs were euthanized by exsanguination under sedation (intramuscular injection of telazol/xylazine/ketamine) and necropsies were performed to obtain tissue samples from the intestinal tract (ileal Peyer’s patches, ileocecal lymph nodes and caecum).

### Sample collection and processing

At days 0, 1, 2, 3 and 7 following wild-type *S.* Typhimurium challenge, swine body temperature was assessed, and blood and faecal samples were obtained. The swine body temperature was monitored using a rectal thermometer (M750, GLA Agricultural Electronics, San Luis Obispo, CA). Blood samples for serum collection were obtained via the jugular vein following the guidelines in NCAH SOP-ARU-0300. Faecal samples were obtained during evacuation for quantitative and qualitative *Salmonella* culture analysis as previously described [7] using XLT-4 medium containing the appropriate antibiotics to select for each strain (50 µg ml^−1^ kanamycin for BBS 866 and 30 µg ml^−1^ nalidixic acid for SB 377). For quantitative and qualitative *Salmonella* culture analysis of tissue samples, processing was performed as described in [7] utilizing XLT-4 medium containing antibiotics as indicated above.

### Interferon-gamma (IFNγ) analysis

The porcine IFNγ ELISA kit (Pierce Biotechnology, Rockford, IL) was used for determination of serum IFNγ levels following *S.* Typhimurium challenge as indicated by the manufacturer’s instructions.

### Statistical analysis of clinical data

Statistical analyses of clinical parameters were performed in GraphPad Prism 5.01 (GraphPad Software, La Jolla, CA). Statistical analysis of body temperatures and IFNγ levels within each treatment group were performed using a repeated measures ANOVA with a Dunnett’s multiple comparison test with comparison to the day 0 control. A two-way repeated measures ANOVA followed by Bonferroni’s multiple comparison test was performed for statistical analysis comparing body temperatures, IFNγ levels and *Salmonella* faecal shedding between vaccinated and mock-vaccinated groups. Statistical analysis of the combined mean body temperature, combined mean IFNγ levels, and tissue and caecal content quantitation was performed using an unpaired *t*-test.

### IDEXX HerdChek swine *Salmonella* antibody assay

To determine the presence of porcine antibodies to *Salmonella* LPS antigen (derived from serogroups B, C1 and D) in serum, the IDEXX HerdChek Swine *Salmonella* Test Kit (IDEXX Europe, Hoofddorp, The Netherlands) was used as previously indicated [3]. A sample's S/P ratio of ≥0.25 was considered positive while <0.25 was considered negative.

### *Salmonella* RNA-Seq

Three biological replicates of strains χ4232 and BBS 866 were prepared for RNA-Seq. A starter culture was diluted 1 : 1000 into 50 ml Luria–Bertani (LB) broth in a 250 ml flask and grown statically for 12 h at 37 °C; a 0.5 ml aliquot from each culture was placed in 1 ml RNAProtect and processed according to the manufacturer’s directions. RNA isolation was performed using the RNeasy Mini Kit (QIAGEN, Germantown, MD) and genomic DNA was removed using Turbo DNase DNA-free (Ambion, Austin, TX). The concentration of the total RNA was determined by using a NanoDrop 8000 spectrophotometer (Thermo Scientific, Wilmington, DE) and RNA quality was assessed using a 2100 Bioanalyzer (Agilent Technologies, Santa Clara, CA). The RiboZero rRNA Removal Kit (Bacteria) was used to deplete rRNA from the total RNA samples (Illumina, San Diego, CA) and the 2100 Bioanalyzer was used to verify that the 16S and 23S peaks were absent. TruSeq Stranded Total RNA Library Prep kits were used to generate libraries that were subsequently sequenced on a HiSeq 2500 using a 100-cycle single-end run (Illumina, San Diego, CA) at the Iowa State University DNA core facility. The sequencing output resulted in an average of 15.4±1.5 million reads per sample. FastQC was used to assess the quality of the reads [8].

### Directional RNA-Seq analysis

CLC Genomics Workbench version 8.5 was used to quality-filter failed reads and to evaluate the sequence data. The reads were mapped separately to the forward and reverse strands of the *S.* Typhimurium strain 798 genome (GenBank: CP003386.1) [9] under the following conditions: 90 % minimum length fraction, 80 % minimum similarity fraction, two maximum mismatches and 10 maximum hits per read [10]. The EdgeR module in CLC Genomics Workbench was used to calculate changes in gene expression for all pairwise comparisons for the sense data. False discovery rate (FDR)-corrected *P*-values ≤0.05 were considered significant.

### RNA isolation and sequencing from porcine blood

Total RNA from whole pig blood preserved in PAXgene blood RNA tubes was isolated using the PAXgene Blood RNA Kit according to the manufacturer’s directions (Qiagen, Valencia, CA) from three pigs at 0 and 2 days post-vaccination. Contaminating DNA was removed by using the Turbo DNA-free kit (Ambion, Life Technologies, Carlsbad, CA). Half of the RNA sample was treated for globin reduction as previously described [11]. The quality and quantity of RNA were assessed using an Agilent 2100 Bioanalyzer (Agilent Technologies, Santa Clara, CA) and a Nanodrop spectrophotometer (Thermo Scientific, Wilmington, DE). Libraries were constructed using the Illumina TruSeq RNA Sample Prep Kit version 2 and were sequenced on an Illumina HiSeq 2500 in a 100-cycle paired-end sequencing run (Illumina, San Diego, CA) at the Iowa State University DNA core facility. The quality of the sequencing reads was assessed using FastQC [8]. Reads were quality trimmed using Trimmomatic [12] and the post-trimming read quality was again assessed with FastQC. Reads were aligned to the *Sus scrofa* genome (Ensembl Sscrofa10.2) with the Bowtie aligner [13] and Samtools was used to convert the Bowtie outputs to a format amenable to gene-wise counting [14]. The read counts per gene were calculated by processing the mapped sequence alignments through HTSeq-count [15]. Finally, DESeq2 [16] was used to specify the analysis model used to perform the differential expression analysis and to extract the results from the desired comparisons. Genes identified as differentially expressed were used as inputs for topGO analysis to further identify the biological processes affected by the experimental factors [17].

### Tissue culture assays

The non-transformed cell line IPEC-J2 (passages 52–57) were derived from porcine jejunal epithelial cells and were a kind gift from Dr Bruce Schultz (Department of Anatomy and Physiology, Kansas State University, Manhattan, KS). Propagation and experimentation with the cell line was performed as previously described with slight modifications [18]. Briefly, the cells were grown and maintained in 50 % DMEM and 50 % Nutrient Mixture F12 (1 : 1 DMEM/F12; Invitrogen Life Technologies, Carlsbad, CA) with 5 % heat-inactivated foetal bovine serum, 5 µg ml^−1^ insulin, 5 µg ml^−1^ transferrin, 5 ng ml^−1^ selenous acid, 5 ng ml^−1^ epidermal growth factor, 100 µg ml^−1^ streptomycin and 100 units ml^−1^ penicillin. The cells were seeded into 24-well cell culture plates (BD Falcon, BD Biosciences, San Jose, CA) at ~2×10^5^ cells per well and maintained in an atmosphere of 5 % CO_2_ at 37 °C for 2 days. Cells were washed thrice with PBS and incubated in DMEM/F12 medium devoid of antibiotics with 18 h statically grown bacterial strains (m.o.i. of 100). Following a 2 h incubation at 37 °C with 5 % CO_2_, cells were washed thrice with PBS and incubated in medium containing 50 µg ml^−1^ gentamicin for 2 h to remove the extracellular bacteria. Cells were washed twice with PBS and 100 µl 1 % Triton X-100 was added to each well for 30 min of incubation at 37 °C. Serial dilutions were performed using the cellular lysates and plated on LB agar medium for overnight growth at 37 °C. The percent invasion was determined by dividing the number of intracellular bacteria by the number of bacteria in the inoculum and multiplying by 100. Three technical replicates were performed for each experiment and statistical analysis was performed in GraphPad Prism 5.01 on three separate experiments using an unpaired *t*-test.

Macrophage survival assays with cell line J774A.1 (ATCC #TIB-67; American Type Culture Collection, Manassas, VA) were performed as described above with the following modifications. Cells were propagated in DMEM with l-glutamine (ATCC#30–2002), 10 % heat-inactivated foetal bovine serum (Invitrogen), 100 µg ml^−1^ streptomycin and 100 units ml^−1^ penicillin. Cells were seeded at ~4×10^6^ cells per well and incubated the following day with the bacterial strains (m.o.i. of 100) for 1 h in medium devoid of antibiotics. Cells were washed thrice with PBS and incubated for 2 h in medium with streptomycin/penicillin at 37 °C, 5 % CO_2_. Cells were harvested with Triton X-100 and percent survival was determined and evaluated for statistical significance as described above.

### Motility assays

Swimming medium was prepared using 0.3 % agar in LB (10 g Bacto tryptone, 5 g Bacto yeast extract, 5 g NaCl, 3 g Bacto agar in 1 L deionized water) [19]. Swim medium was autoclaved and placed in a 56 °C water bath. After temperature equilibration, medium was pipetted into plates, covered and allowed to solidify for 30 min. Once the agar was solidified, Petri plate lids were removed and the motility medium was allowed to dry for 10 min in a biological safety cabinet prior to inoculation with the bacterial culture. Strains were grown overnight in LB broth at 37 °C with shaking. Overnight cultures were diluted 1 : 200 in LB broth and grown at 37 °C with shaking to OD_600_=0.3–0.4. A 5 µl sample from the sub-culture was placed in the centre of each plate and the plate was covered and allowed to sit for 5 min. The plates were placed in a 37 °C humid incubator for 8 h prior to measurement of the motility zone diameter. Four technical replicates were performed for each condition and the experiment was repeated three times. Motility differences between strains was assessed using an unpaired *t*-test.

## Results and discussion

### Vaccination prevents *S.* Typhimurium-induced elevation in body temperature (fever)

**Fig. 1.**
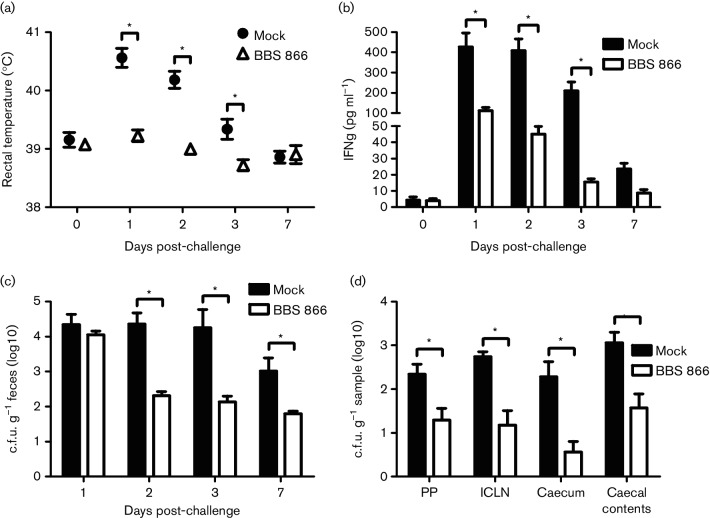
Average body temperature, serum IFNγ levels and *S.* Typhimurium faecal shedding and tissue colonization following swine challenge. At 9 weeks of age, mock-vaccinated and BBS 866-vaccinated swine (*n*=10 per group) were intranasally challenged with PBS containing 2×10^8^ c.f.u. wild-type *S.* Typhimurium UK1. Body temperature (a) and serum IFNγ levels (b) were monitored at 0, 1, 2, 3 and 7 days following challenge. *S.* Typhimurium faecal shedding was determined on days 1, 2, 3 and 7 following challenge (c). Colonization of gastrointestinal tissues (Peyer’s patch, PP; ileocecal lymph nodes, ICLN; and caecum] and caecal content by *S.* Typhimurium was determined at day 7 post-challenge (d). The data symbols and bars denote the mean and the error bars indicate the standard error of the mean (sem). *Statistically significant differences between groups on the individual days.

Previously, we described the rational design and construction of an attenuated *S.* Typhimurium vaccine strain (BBS 866) to confer cross-protection to a broad range of *Salmonella* serovars [5]. Following vaccination of swine with BBS 866 and challenge with virulent *S.* Choleraesuis, we demonstrated reduced colonization and systemic disease as well as DIVA. To determine the protection of BBS 866 against the common food-borne pathogen *S.* Typhimurium, 4-week-old piglets were administered an intranasal inoculation of either 1 ml PBS (mock-vaccinated) or PBS containing 1.0×10^10^ c.f.u. BBS 866 vaccine strain. Two weeks later, pigs received a booster of either PBS or PBS containing 4.0×10^9^ c.f.u. BBS 866. At 9 weeks of age, all pigs were intranasally challenged with 1 ml PBS containing 2×10^8^ c.f.u. virulent *S.* Typhimurium UK1 strain (SB 377). Following challenge with wild-type *S.* Typhimurium UK1, the average swine body temperature at days 1, 2 and 3 post-challenge was significantly lower (*P*<0.001, *P*<0.001 and *P*<0.01, respectively) in vaccinated pigs compared to mock-vaccinated pigs (Fig. 1a). In mock-vaccinated pigs, the body temperature was significantly elevated (*P*<0.001) at days 1 and 2 post-challenge compared to day 0. Furthermore, the combined mean body temperature for days 1, 2 and 3 post-challenge was significantly higher (*P*<0.0001) in mock-vaccinated pigs (40.03 °C) compared to vaccinated pigs (38.98 °C). Based on the body temperature assessment, the data indicate that vaccination of swine with BBS 866 reduces disease severity due to virulent *S.* Typhimurium UK1.

### Vaccination reduces IFNγ response in wild-type *S.* Typhimurium-challenged swine

A Th1 immune response is generated against intracellular bacterial pathogens, including *Salmonella*, and is characterized by the production of the cytokine IFNγ. Thus, serum IFNγ levels were measured to assess the Th1 immune response induced by the *Salmonella* challenge. The serum IFNγ levels in mock-vaccinated swine were significantly greater (*P*<0.001) at days 1, 2 and 3 post-challenge with virulent *S.* Typhimurium compared to vaccinated pigs (Fig. 1b). In mock-vaccinated pigs, the IFNγ levels at days 1, 2 and 3 post-challenge were significantly higher (*P*<0.001, *P*<0.001 and *P*<0.01, respectively) compared to day 0. The IFNγ levels were significantly elevated (*P*<0.001) at days 1 and 2 post-challenge in vaccinated pigs compared to day 0. The combined mean IFNγ levels for days 1, 2 and 3 post-challenge were significantly lower (*P*<0.0001) in vaccinated pigs (57.8 pg ml^−1^) compared to mock-vaccinated pigs (349.5 pg ml^−1^). Although BBS 866-vaccinated pigs had elevated IFNγ levels at days 1 and 2 post-challenge, this finding was significantly lower than the mock-vaccinated swine and was not accompanied by an increase in body temperature. Thus, an immune response was activated in both of the challenged groups, but only mock-vaccinated swine presented with pyrexia.

### Vaccination reduces *Salmonella* faecal shedding in wild-type *S.* Typhimurium-challenged swine

Following challenge with wild-type *S.* Typhimurium, faecal shedding of *Salmonella* was significantly reduced by up to 2 logs in vaccinated pigs at days 2, 3 and 7 post-challenge (*P*<0.001, *P*<0.001 and *P*<0.05, respectively) compared to mock-vaccinated pigs (Fig. 1c). Prior research by our group has correlated higher porcine IFNγ levels with greater *Salmonella* shedding in swine faeces [20, 21]. Furthermore, pigs that persistently shed greater levels of *Salmonella* also had a longer duration of pyrexia [21]. Our previous correlation of greater faecal shedding of *Salmonella* in pigs with both increased IFNγ levels and body temperature is supported by the results we obtained in this study; mock-vaccinated swine shed significantly greater levels of *S.* Typhimurium while exhibiting both pyrexia and higher IFNγ levels compared to BBS 866-vaccinated swine. Recently, Snary *et al*. performed a farm-to-consumption quantitative microbiological risk assessment (QMRA) that demonstrated pigs shedding ≥10^4^ c.f.u. *Salmonella* per gram of faeces account for the vast majority of human disease risk from pork consumption [22]. At days 2 and 3 post-challenge with *S.* Typhimurium, significantly more mock-vaccinated swine (six versus zero for both days; *P*=0.0108) were shedding ≥10^4^ c.f.u. *Salmonella* per gram of faeces compared to BBS 866-vaccinated pigs. Vaccination with BBS 866 would mitigate the risk of *Salmonella* in pork by decreasing the on-farm levels of this food safety pathogen.

At day 7 post-challenge, all pigs were euthanized and tissues were obtained during necropsy. The c.f.u. g^−1^ of tissue was significantly reduced by up to 2 logs in vaccinated pigs for the Peyer’s patch, ileocecal lymph node and caecum (*P*=0.0081, *P*=0.0003, and *P*=0.0006, respectively) compared to mock-vaccinated swine (Fig. 1d). In vaccinated swine, the c.f.u. g^−1^ caecal content was significantly lower (*P*=0.0016) compared to mock-vaccinated pigs (Fig. 1d). Together, the reduction in *Salmonella* tissue colonization and faecal shedding in the vaccinated pigs demonstrate the ability of the BBS 866 vaccine to decrease *Salmonella* in swine.

### BBS 866 is a DIVA vaccine

**Fig. 2.**
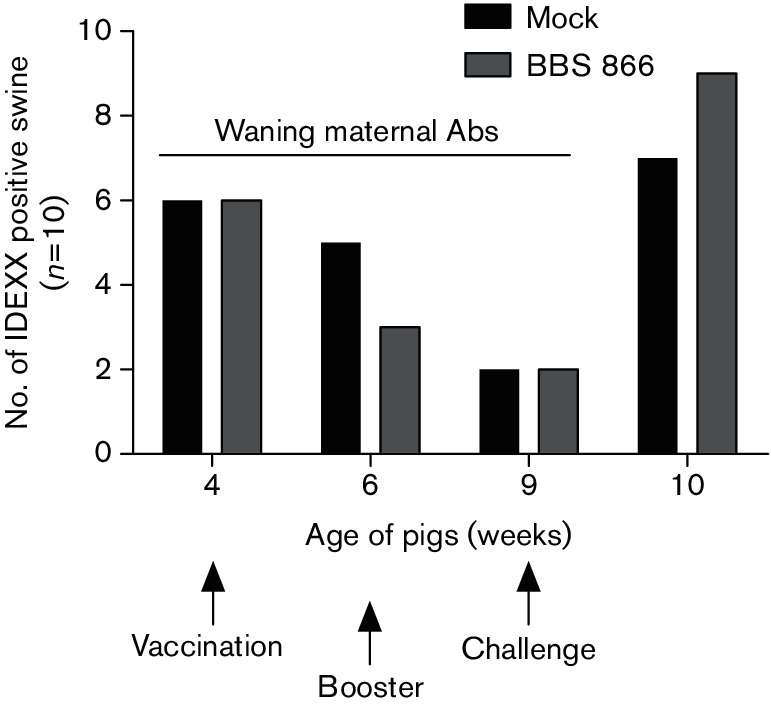
Analysis of porcine serum antibodies to *Salmonella* LPS following *Salmonella* vaccination and challenge of pigs. The number of pigs with serum containing antibodies to *Salmonella* LPS at 4 weeks (vaccination), 6 weeks (booster vaccination), 9 weeks (challenge) and 10 weeks (7 days post-challenge) of age for mock-vaccinated and BBS 866-vaccinated pigs. The number of positive swine at each time point are shown. All pigs were challenged with virulent *S.* Typhimurium at 9 weeks of age. An S/P ratio of ≥0.25 was considered positive and <0.25 was considered negative.

Twenty offspring from four sows comprised the groups for the vaccine trial. Prior to farrowing, we screened the four sows for active shedding of *Salmonella* in their faeces and serum antibodies to *Salmonella* LPS. None of the sows were actively shedding *Salmonella* and three of the sows were positive for serum antibodies to *Salmonella* LPS; serum volume was not sufficient to screen the fourth sow for anti-*Salmonella* LPS antibodies. At 4 weeks of age (day of vaccination), piglet serum was obtained to determine the presence of antibodies to *Salmonella* LPS. A subset of piglets from three of the sows was positive for serum antibodies to *Salmonella* LPS in both the mock-vaccinated and vaccinated groups (Fig. 2). By 9 weeks of age (day of virulent *S.* Typhimurium challenge), serum antibodies to *Salmonella* LPS had waned in a majority of the piglets, suggesting that the presence of the antibodies was maternally derived. A similar number of piglets with serum antibodies to LPS had declined in both groups by 9 weeks of age, indicating that vaccination did not induce an antibody response to *Salmonella* LPS. However, 1 week following challenge with virulent *S.* Typhimurium, a majority of piglets in both the mock-vaccinated and vaccinated groups seroconverted for the presence of antibodies to *Salmonella* LPS. This indicates, as previously demonstrated, that vaccination with BBS 866 does not prevent an immune response against *Salmonella* LPS. Collectively, these results signify that BBS 866 can be used during swine herd management for DIVA [5].

### Gene expression differences between the vaccine and parental *S*. Typhimurium strains

**Fig. 3.**
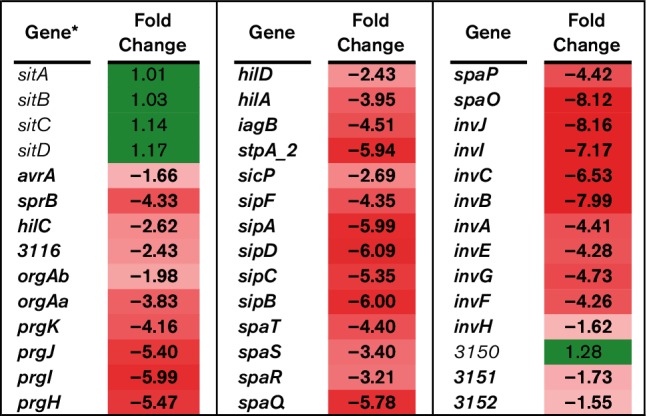
RNA-Seq analysis of SPI-1 genes. RNA-Seq identified the fold-change in expression of SPI-1 genes comparing BBS 866 to χ4232. Gene expression is significantly different (FDR-adjusted *P*<0.05) between strains if the gene and its corresponding fold-change in expression are indicated in bold. Green denotes increased gene expression and red indicates decreased gene expression; the intensity of the colour highlights greater change. *Gene with only a number assignment represent the UNM798 gene designation.

*Salmonella* virulence results from a complex interaction between the bacterial pathogen and the eukaryotic host, with multiple regulons participating at various points in the infection cycle. Previously, we demonstrated that BBS 866 was attenuated for virulence in swine [5]. In this study, RNA-Seq was utilized to determine gene expression differences between BBS 866 and its virulent parent strain χ4232 following 12 h of static incubation at 37 °C to replicate culture conditions used for animal vaccination. Between BBS 866 and χ4232, 1579 genes were differentially regulated based on FDR-corrected *P*-values<0.05, including numerous genes associated with virulence. *Salmonella* pathogenicity island 1 (SPI-1) is associated with invasion of eukaryotic cells; 37 of 42 SPI-1 genes were significantly downregulated in BBS 866 compared to χ4232 (Fig. 3). Since a majority of SPI-1 genes were downregulated and because invasion is an integral phenotype for *Salmonella* virulence, we conducted *in vitro* assays to determine whether BBS 866 had decreased invasion of the porcine-derived IPEC-J2 cell line. The percent invasion of IPEC-J2 by BBS 866 was 0.0285 %, a significant reduction (*P*=0.0162) of ~70-fold compared to χ4232 with an invasion rate of 2.062 %. Our invasion assay results using IPEC-J2 cells is consistent with the observed reduction of invasion gene expression in BBS 866 compared to χ4232.

**Fig. 4.**
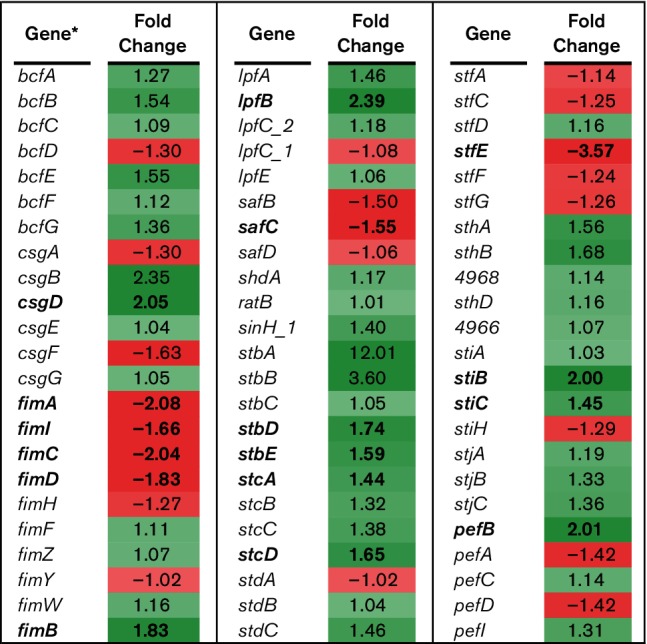
RNA-Seq analysis of attachment genes. RNA-Seq identified the fold-change in expression of genes associated with bacterial attachment comparing BBS 866 to χ4232. Gene expression is significantly different (FDR-adjusted *P*<0.05) between strains if the gene and its corresponding fold-change in expression are indicated in bold. Green denotes increased gene expression and red indicates decreased gene expression; the intensity of the colour highlights greater change. *Gene with only a number assignment represent the UNM798 gene designation.

Bacterial invasion requires intimate contact with eukaryotic cells, and mediators of adhesion and attachment influence the physical interaction between *Salmonella* and its host. Of the seven SPI-4 genes in χ4232, five were significantly downregulated in BBS 866 compared to the parental strain including genes encoding the SiiE adhesin and the type III secretion apparatus for SiiE export. The reduction in gene expression for *siiBCDEF* was approximately 54-, 40-, 307-, four- and five-fold, respectively (data not shown). For the 13 fimbrial operons associated with bacterial attachment, 10 genes were significantly upregulated and six were significantly downregulated in BBS 866 compared to χ4232 (Fig. 4). Five of the 10 *fim* genes required for regulation and production of type 1 fimbriae were differentially regulated; four significantly downregulated (*fimAICD*) and one significantly upregulated (*fimB*). An *S.* Typhimurium *fimA* mutant was previously shown to have decreased colonization of the swine intestine compared to wild-type *S.* Typhimurium [23]. Based on these results, a reduction in adhesion or attachment of BBS 866 to epithelial cells could have an overall impact on the invasion efficiency for IPEC-J2 cells and swine colonization.

**Fig. 5.**
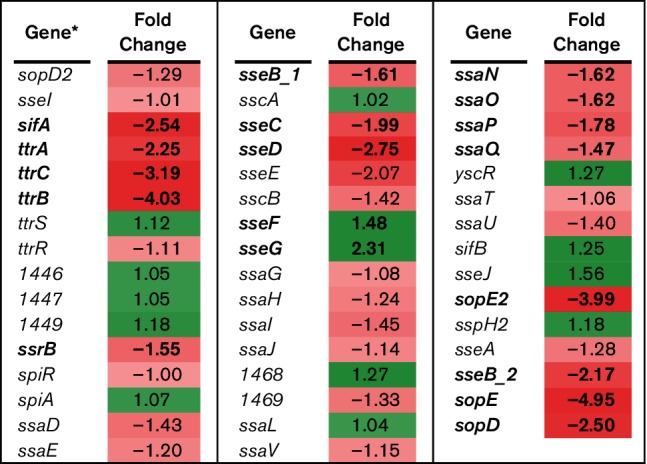
RNA-Seq analysis of SPI-2 genes. RNA-Seq identified the fold-change in expression of SPI-2 genes comparing BBS 866 to χ4232. Gene expression is significantly different (FDR-adjusted *P*<0.05) between strains if the gene and its corresponding fold-change in expression are indicated in bold. Green denotes increased gene expression and red indicates decreased gene expression; the intensity of the colour highlights greater change. *Gene with only a number assignment represent the UNM798 gene designation.

Intracellular replication and survival of *Salmonella* requires the SPI-2 regulon. Of 47 SPI-2 genes, 16 were significantly downregulated and two significantly upregulated in BBS 866 compared to χ4232 (Fig. 5). We performed macrophage survival assays using cell line J774A.1 and strains BBS 866 and χ4232. A significant difference in macrophage survival comparing BBS 866 and χ4232 was not observed (data not shown).

**Fig. 6.**
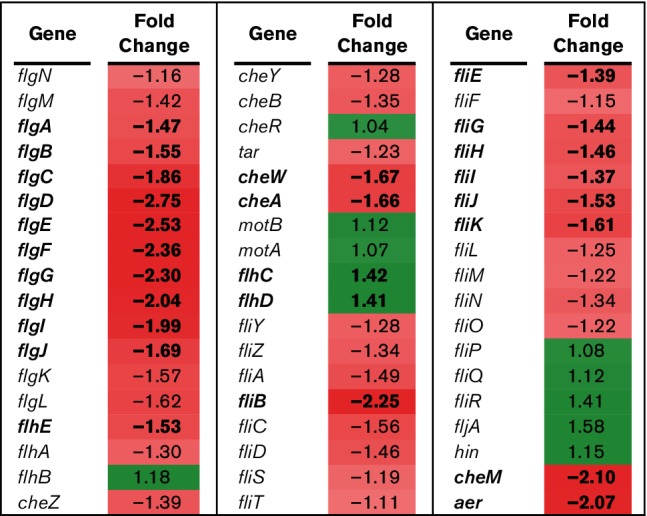
RNA-Seq analysis of motility genes. RNA-Seq identified the fold-change in expression of genes associated with bacterial motility comparing BBS 866 to χ4232. Gene expression is significantly different (FDR-adjusted *P*<0.05) between strains if the gene and its corresponding fold-change in expression are indicated in bold. Green denotes increased gene expression and red indicates decreased gene expression; the intensity of the colour highlights greater change.

Bacterial motility has been associated with *Salmonella* pathogenicity. Of 54 motility related genes, 22 were significantly downregulated and two significantly upregulated in BBS 866 compared to χ4232 (Fig. 6). The average diameter of BBS 866 on motility swim medium containing 0.3 % agar was 14.25 mm following 8 h of incubation, a significant reduction (*P*<0.0002) compared to χ4232 with a diameter of 54.67 mm. The motility phenotype is in agreement with the significant downregulation of motility gene expression in BBS 866.

**Fig. 7.**
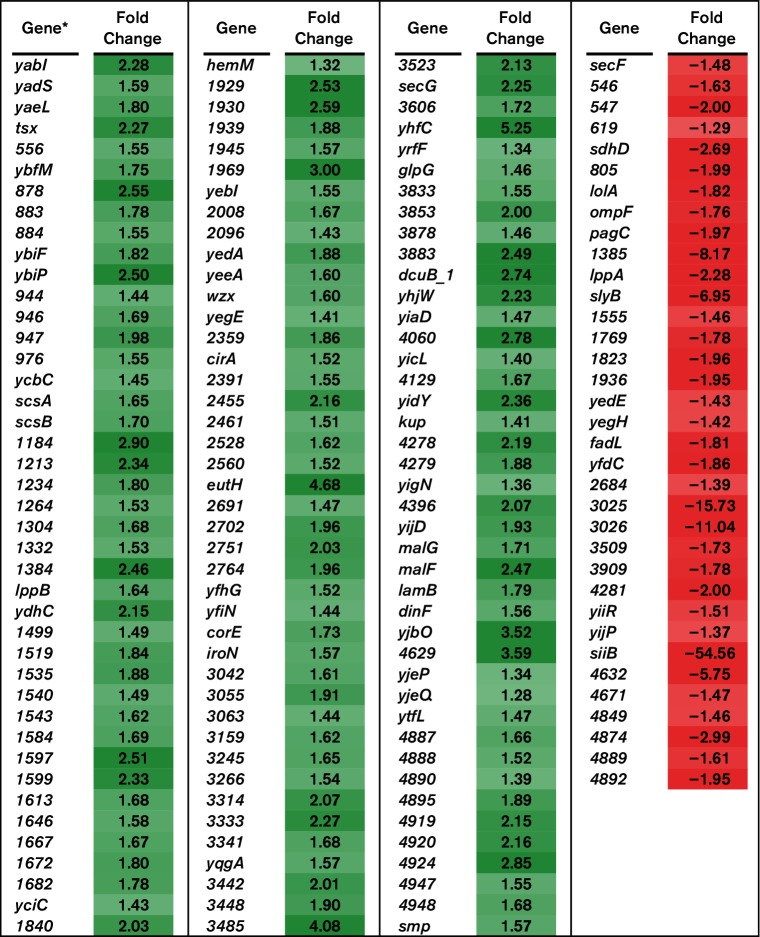
RNA-Seq analysis of membrane-associated genes. RNA-Seq identified the fold-change in expression of membrane-associated genes comparing BBS 866 to χ4232. Gene expression is significantly different (FDR-adjusted *P*<0.05) between strains for all of the genes listed. Green denotes increased gene expression and red indicates decreased gene expression; the intensity of the colour highlights greater change. *Gene with only a number assignment represent the UNM798 gene designation.

The BBS 866 strain contains deletions in the small regulatory RNAs *invR*, *micA*, *omrA*, *omrB* and *rybB*. These sRNAs post-transcriptionally regulate mRNAs encoding outer membrane proteins. Genes classified as encoding membrane proteins included 382 genes, of which 161 were differentially regulated. For the 161 differentially regulated genes encoding membrane proteins, 126 were significantly upregulated and 35 were significantly downregulated in BBS 866 compared to χ4232 (Fig. 7). Specifically, gene expression of *tsx*, *lamB* and *cirA* was increased and their expression is regulated by the sRNAs *rybB*, *micA* and *omrAB*, respectively [24, 25]. The increase in gene expression for a subset of membrane proteins produced by BBS 866 may present the host immune system with conserved antigens during vaccination that confer protection against challenge with various serovars of *Salmonella*. Indeed, data presented herein and in our previous vaccine trial indicates that vaccination of swine with BBS 866 provides protection against both homologous and heterologous serovars of *Salmonella*, as demonstrated for Typhimurium and Choleraesuis, respectively [5].

### Transcriptional response of swine to *S*. Typhimurium vaccine

The porcine gene expression response to the *Salmonella* vaccine strain was evaluated by RNA-Seq using total RNA isolated from peripheral blood of the pigs before and 2 days after vaccination; all changes are relative to the pre-vaccination sample (day 2/day 0). Vaccination resulted in the differential expression of 830 genes (FDR<0.1). Using topGo enrichment analysis for gene ontology (GO) terms [17], we identified the biological processes involved in a variety of systems, especially the immune and inflammatory response (Table S1, available in the online Supplementary Material). Porcine genes that our research group had previously described as *Salmonella*-responsive [21, 26] were upregulated, such as IL1RAP (interleukin 1 receptor accessory protein), TNFAIP6 (tumour necrosis factor alpha-induced protein 6) and TGM1 (transglutimase 1) (Table 1). Because our previous transcriptional analyses of the porcine response to the parental, wild-type *S*. Typhimurium strain utilized Affymetrix Genechip microarrays, a direct comparison to our current RNA-Seq transcriptional data of the porcine response to the *S*. Typhimurium vaccine is difficult due to the different technologies employed for each study. However, we noted from the datasets that both the vaccine and wild-type strains induced significant transcriptional changes in the swine immune response, although a diminution in the pigs' response to the vaccine strain (current study) compared to pigs that received the parental strain [21, 26] was observed for some genes, including TCN1 (eightfold versus 246-fold), KCNJ15 (13-fold versus 92-fold) and TGM3 (22-fold versus 68-fold). A gene that we have repeatedly observed as upregulated in *Salmonella*-challenged pigs is CXCL10, also known as interferon gamma-induced protein 10 (IP-10), which binds the CXCR3 receptor to cause pleiotropic effects in chemotaxis, apoptosis and angiostasis [27]. CXCL10 has been identified as a biomarker for severity prediction of various diseases [28, 29]. Also notable is that porcine genes identified with the greatest fold-change following vaccination are involved in regulating the inflammatory response (IL22RA2, inhibitor of IL22) and suppressing the immune system (PDL1, also known as CD274), which may contribute to the absence of clinical symptoms (fever and diarrhoea) in the vaccinated pigs. Collectively, the data indicates that vaccination with BBS 866 induces transcriptional alterations in a variety of porcine genes including immune response genes.

**Table 1.** Top 50 differentially expressed porcine genes with the highest fold-change in response to the *S*. Typhimurium vaccine strain BBS 866 (2 days post-vaccination/0 days post-vaccination)

Log2 fold change	Fold change	*P* value	False discovery rate	Ensembl*	Gene symbol	Gene description	Source
10.31	1272.60	2.14E-05	7.87E-04	03601	HCRTR1	Hypocretin (orexin) receptor 1	HGNC Symbol; Acc:HGNC:4848
6.13	69.99	2.26E-24	1.49E-21	05211	PDL1	CD274 molecule (CD274), mRNA (*Sus scrofa*)	RefSeq mRNA; Acc:NM_001025221
6.05	66.07	9.66E-35	2.70E-31	04158	IL22RA2	Interleukin 22 receptor, alpha 2	HGNC Symbol; Acc:HGNC:14901
5.73	53.06	4.78E-10	5.24E-08	23 525	TMEM26	Transmembrane protein 26 (TMEM26), mRNA (*Sus scrofa*)	RefSeq mRNA; Acc:NM_001244582
5.69	51.53	1.01E-06	5.53E-05	07710	CHREBP	Uncharacterized protein	UniProtKB/TrEMBL; Acc:F1RJN3
4.87	29.29	5.48E-05	1.78E-03	06451	PCD1B	CD1B antigen (PCD1B), mRNA (*Sus scrofa*)	RefSeq mRNA; Acc:NM_001097491
4.78	27.51	6.30E-06	2.74E-04	08771	C4orf19	Chromosome 4 ORF 19	HGNC Symbol; Acc:HGNC:25618
4.47	22.15	2.52E-32	4.71E-29	26 043	TGM3	Transglutaminase 3	HGNC Symbol; Acc:HGNC:11779
4.45	21.92	2.08E-12	3.37E-10	08977	CXCL10	Chemokine (C-X-C motif) ligand 10 (CXCL10), mRNA (*Sus scrofa*)	RefSeq mRNA; Acc:NM_001008691
4.36	20.53	6.22E-31	8.69E-28	09469		Uncharacterized protein	UniProtKB/TrEMBL; Acc:F1RHE5
4.19	18.31	6.51E-03	9.19E-02	04236		Uncharacterized protein	UniProtKB/TrEMBL; Acc:F1SF58
4.08	16.92	1.75E-14	3.92E-12	21 206	IL1RAP	Interleukin 1 receptor accessory protein	HGNC Symbol; Acc:HGNC:5995
4.07	16.74	3.37E-03	5.62E-02	16 175	MREG	Melanoregulin	HGNC Symbol; Acc:HGNC:25478
3.99	15.84	2.06E-03	3.83E-02	10 505	DNTT	DNA nucleotidylexotransferase	HGNC Symbol; Acc:HGNC:2983
3.95	15.41	1.45E-03	2.86E-02	15 716	MARCO	Macrophage receptor with collagenous structure	HGNC Symbol; Acc:HGNC:6895
3.87	14.62	5.10E-05	1.69E-03	19 886	SNORA27	Small nucleolar RNA SNORA27	RFAM; Acc:RF00443
3.87	14.61	3.10E-03	5.28E-02	22 500	IL20RB	Interleukin 20 receptor beta	HGNC Symbol; Acc:HGNC:6004
3.74	13.32	9.18E-08	6.42E-06	05210		Uncharacterized protein	UniProtKB/TrEMBL; Acc:F1SK52
3.70	12.96	7.49E-14	1.47E-11	11 712	P2RY14	Purinergic receptor P2Y, G-protein coupled, 14	HGNC Symbol; Acc:HGNC:16442
3.67	12.70	1.35E-08	1.18E-06	12 066	KCNJ15	Potassium channel, inwardly rectifying subfamily J, member 15	HGNC Symbol; Acc:HGNC:6261
3.67	12.69	5.56E-04	1.30E-02	08103		Uncharacterized protein	UniProtKB/TrEMBL; Acc:F1SU88
3.53	11.57	4.78E-35	1.78E-31	01561	ETV7	ETS variant 7	HGNC Symbol; Acc:HGNC:18160
3.45	10.92	9.34E-05	2.79E-03	13 788		Uncharacterized protein	UniProtKB/TrEMBL; Acc:F1SCE1
3.44	10.88	3.80E-08	2.93E-06	00649	CLEC1A	C-type lectin domain family 1, member A	HGNC Symbol; Acc:HGNC:24355
3.41	10.62	2.94E-20	1.73E-17	17 721	CCL8	Chemokine (C-C motif) ligand 8 (CCL8), mRNA (*Sus scrofa*)	RefSeq mRNA; Acc:NM_001164515
3.39	10.51	4.30E-19	2.00E-16	07557	MICALL2	MICAL-like 2	HGNC Symbol; Acc:HGNC:29672
3.37	10.36	2.91E-31	4.64E-28	01993	TGM1	Transglutaminase 1	HGNC Symbol; Acc:HGNC:11777
3.35	10.21	6.47E-03	9.16E-02	26 948		Uncharacterized protein	UniProtKB/TrEMBL; Acc:I3LJY7
3.27	9.67	1.28E-30	1.59E-27	10 445	ANKRD22	Ankyrin repeat domain 22	HGNC Symbol; Acc:HGNC:28321
3.17	9.00	2.51E-03	4.47E-02	25 754	ZGLP1	Zinc finger, GATA-like protein 1	HGNC Symbol; Acc:HGNC:37245
3.16	8.94	1.00E-15	3.11E-13	28 875	HAVCR2	Hepatitis A virus cellular receptor 2	HGNC Symbol; Acc:HGNC:18437
3.12	8.71	4.88E-08	3.69E-06	03525	C1QC	Complement component 1, q subcomponent, C chain (C1QC), mRNA (*Sus scrofa*)	RefSeq mRNA; Acc:NM_001244286
3.09	8.53	5.31E-05	1.73E-03	16 244	COL4A4	Collagen, type IV, alpha 4	HGNC Symbol; Acc:HGNC:2206
3.09	8.49	9.90E-06	4.04E-04	03524	C1QA	Complement component 1, q subcomponent, A chain (C1QA), mRNA (*Sus scrofa*)	RefSeq mRNA; Acc:NM_001003924
3.03	8.20	4.89E-03	7.43E-02	01544	TEAD3	TEA domain family member 3	HGNC Symbol; Acc:HGNC:11716
3.02	8.11	6.80E-10	7.05E-08	04444	FAM26F	Family with sequence similarity 26, member F	HGNC Symbol; Acc:HGNC:33391
3.00	8.02	1.90E-03	3.57E-02	00866	MYBPC1	Myosin binding protein C, slow type	HGNC Symbol; Acc:HGNC:7549
3.00	8.01	1.49E-19	7.23E-17	06763	BCL2L15	BCL2-like 15	HGNC Symbol; Acc:HGNC:33624
3.00	8.01	1.72E-05	6.48E-04	05617	STXBP1	Syntaxin binding protein 1	HGNC Symbol; Acc:HGNC:11444
2.97	7.82	2.29E-03	4.17E-02	28 525	LOC733603	Serum amyloid A2	RefSeq mRNA; Acc:NM_001044552
2.97	7.81	6.89E-04	1.54E-02	14 996	CASP12	Caspase 12 (gene/pseudogene)	HGNC Symbol; Acc:HGNC:19004
2.96	7.81	9.70E-27	9.04E-24	24 867	ISG20	Interferon stimulated exonuclease gene 20 kDa	HGNC Symbol; Acc:HGNC:6130
2.96	7.79	5.56E-03	8.14E-02	13 116	TCN1	Transcobalamin I	RefSeq mRNA; Acc:NM_001062
2.96	7.78	2.92E-04	7.51E-03	26 832	CSF1	Colony stimulating factor 1 (macrophage) (CSF1), mRNA (*Sus scrofa*)	RefSeq mRNA; Acc:NM_001244523
2.95	7.75	1.23E-04	3.54E-03	28 486		Uncharacterized protein	UniProtKB/TrEMBL; Acc:I3LT32
2.95	7.74	7.91E-07	4.63E-05	03482	PADI3	Peptidyl arginine deiminase, type III	HGNC Symbol; Acc:HGNC:18337
2.94	7.68	2.29E-09	2.23E-07	19 937	SNORA19	Small nucleolar RNA SNORA19	RFAM; Acc:RF00413
2.92	7.59	4.63E-11	6.56E-09	07007	IDO1	Indoleamine 2,3-dioxygenase 1	HGNC Symbol; Acc:HGNC:6059
2.90	7.46	1.76E-03	3.34E-02	08312	DYSF	Dysferlin	HGNC Symbol; Acc:HGNC:3097
2.88	7.37	1.91E-05	7.13E-04	16 396	TNFAIP6	Tumor necrosis factor, alpha-induced protein 6 (TNFAIP6), mRNA (*Sus scrofa*)	RefSeq mRNA; Acc:NM_001159607

The top 50 differentially expressed genes were all up-regulated.

*The Ensembl number is proceeded by ENSSSCG000000.

### Conclusion

Vaccination of swine with BBS 866 reduces disease, pathogen shedding and gastrointestinal colonization by virulent *S.* Typhimurium. Previously, we demonstrated that vaccination cross-protects pigs against systemic disease due to *S.* Choleraesuis. Thus, the DIVA vaccine provides swine with protection against both homologous and heterologous serovars of *Salmonella* that present a wide disease spectrum from asymptomatic colonization to severe illness and death. As part of a comprehensive swine management strategy, vaccination of pigs with BBS 866 will protect animal health, reduce shedding into the environment and enhance food safety by limiting the transmission of this important food-borne pathogen from pen to plate.

### Disclosure statements

US and European patent applications have been filed by the US Department of Agriculture for *S.* Typhimurium BBS 866 with B. L. B. and S. M. D. B. indicated as co-inventors on the patent applications.

Mention of trade names or commercial products in this article is solely for the purpose of providing specific information and does not imply recommendations or endorsement by the US Department of Agriculture.

USDA is an equal opportunity provider and employer.

## Supplementary Data

152 Supplementary File 1
