# Supplementary material for: Salmonella DIVA vaccine reduces disease, colonization and shedding due to virulent S. Typhimurium infection in swine
Source: J Med Microbiol. 2017 May 18;66(5):651–61. doi: 10.1099/jmm.0.000482 (PMC5817229; doi:10.1099/jmm.0.000482)
Supplement: Supplementary File 1 [file jmm-66-651-s001.pdf]

Table S1. Top 30 GO terms enriched for biological processes as detected by Fisher's Exact Test at p < 0.01 and the weight01 algorithm of the Biocondutor topGO package.

GO.ID	Term	Annotated*	Significant†	Expected‡	Fisher.weight01
GO:0006954	inflammatory response	111	56	16.43	7.40E-10
GO:0007186	G-protein coupled receptor signaling pathway	122	45	18.06	3.90E-08
GO:0050830	defense response to Gram-positive bacteria	13	11	1.92	4.00E-08
GO:0070098	chemokine-mediated signaling pathway	17	12	2.52	3.00E-07
GO:0006955	immune response	274	103	40.56	3.40E-07
GO:0071222	cellular response to lipopolysaccharide	37	23	5.48	6.90E-06
GO:0051607	defense response to virus	60	21	8.88	7.60E-06
GO:0046330	positive regulation of JNK cascade	24	13	3.55	4.00E-05
GO:0031663	lipopolysaccharide-mediated signaling pathway	16	11	2.37	4.00E-05
GO:0032722	positive regulation of chemokine production	9	7	1.33	4.10E-05
GO:0050715	positive regulation of cytokine secretion	21	11	3.11	5.80E-05
GO:0050729	positive regulation of inflammatory response	19	12	2.81	6.10E-05
GO:0006956	complement activation	8	6	1.18	0.00022
GO:0042108	positive regulation of cytokine biosynthetic process	16	9	2.37	0.00028
GO:0045766	positive regulation of angiogenesis	28	12	4.14	0.00031
GO:0033032	regulation of myeloid cell apoptotic process	6	5	0.89	0.00037
GO:0048246	macrophage chemotaxis	6	5	0.89	0.00037
GO:0045064	T-helper 2 cell differentiation	6	5	0.89	0.00037
GO:0045980	negative regulation of nucleotide metabolic process	6	5	0.89	0.00037
GO:0034142	toll-like receptor 4 signaling pathway	6	5	0.89	0.00037
GO:1902930	regulation of alcohol biosynthetic process	12	7	1.78	0.00047
GO:0045071	negative regulation of viral genome replication	15	8	2.22	0.00054
GO:0002698	negative regulation of immune effector process	25	11	3.7	0.00056
GO:0002474	antigen processing/presentation of peptide antigen via MHC class I	12	7	1.78	0.0006
GO:0019221	cytokine-mediated signaling pathway	85	35	12.58	0.00067
GO:0050778	positive regulation of immune response	117	46	17.32	0.00092
GO:0050727	regulation of inflammatory response	61	30	9.03	0.00096
GO:0002429	immune response-activating cell surface receptor signaling	45	13	6.66	0.00111
GO:0015909	long-chain fatty acid transport	7	5	1.04	0.00113
GO:0042088	T-helper 1 type immune response	7	5	1.04	0.00113

*Annotated - Number of genes mapped to the GO term from the annotated list of Sus scrofa genes in the test

†Significant - Number of genes mapped to the GO term in the differentially expressed gene list

‡Expected - Number of genes expected to be mapped to the GO term if differentially expressed genes are randomly distributed over all GO terms
